# The molecular mechanisms underlying hydrogen peroxide regulating free-living conchocelis maturation of *Pyropia haitanensis*

**DOI:** 10.3389/fpls.2026.1979927

**Published:** 2026-10-12

**Authors:** Rong He, Wenfei Xian, Yan Xu, Dehua Ji, Chaotian Xie, Wenlei Wang

**Affiliations:** 1 Fisheries College, Jimei University, Xiamen, China; 2 State Key Laboratory of Mariculture Breeding, Fisheries College, Jimei University, Ningde, China; 3 Key Laboratory of Healthy Mariculture for the East China Sea, Ministry of Agriculture, Xiamen, China

**Keywords:** conchocelis maturation, H2O2 signal, multi-omics analysis, *Pyropia haitanensis*, regulatory mechanism

## Abstract

The transition from conchocelis to conchosporangial branches is not only a key stage in the life cycle of *Pyropia/Porphyra* but also directly affects the yield and quality of economically important species such as *Pyropia haitanensis*. However, the molecular mechanisms underlying the maturation of *P. haitanensis* filaments remain poorly understood. Hydrogen peroxide (H_2_O_2_), as an important signaling molecule, plays a significant role in algae development. This study demonstrated that treatment with the H_2_O_2_ scavenger DMTU (500 μmol/L) significantly reduced H_2_O_2_ and O_2_^-^ levels within the algae, affecting the activity of antioxidant enzymes and the content of antioxidant substances. This disrupted the ROS balance, inhibited key pathways such as photosynthesis and nitrogen metabolism, and triggered dysregulation in downstream metabolite synthesis. Consequently, the levels of most amino acids and their derivatives, nucleotides, and lipids were decreased. This diverted energy and resources from the “maturation program” towards a “stress-response program,” thereby delaying conchocelis maturation. Notably, this inhibitory effect was reversible following DMTU removal. When unfavorable conditions necessitate delayed maturation induction, intervention with DMTU could be applied to effectively reduce the risk of conchospore “abortion”. In summary, H_2_O_2_ is a critical signaling molecule regulating the maturation of *P. haitanensis* conchocelis. This study provides a theoretical basis for elucidating the molecular regulatory network governing conchocelis maturation in *Pyropia/Porphyra* and achieving precise regulation of synchronized maturation.

## 1 Introduction

The life cycle of *Pyropia/Porphyra* exhibits an alternation of generations, comprising a haploid thallus (gametophyte) and a diploid filamentous stage (conchocelis or sporophyte) (Lin et al., 2021). This life cycle strategy serves as a key adaptive mechanism for survival in the highly variable intertidal environment. The life cycle proceeds as follows: the thallus normally matures in autumn and winter, releasing male and female gametes that form diploid carpospores upon fertilization. The released carpospores bore into shells and develop into conchocelis. The conchocelis mature and differentiate into conchosporangial branches in summer, which subsequently release conchospores. These conchospores then undergo meiosis to develop into the haploid gametophytic thallus. Within this alternation of generations, the transition from filamentous conchocelis to conchosporangial branches represents a critical developmental stage. Rigorous investigation into conchocelis developmental mechanisms not only strengthens the theoretical foundation of conchocelis ontogeny but also yields practical technical solutions for enhancing the efficiency and reliability of *Pyropia/Porphyra* seedling production, thereby contributing to sustainable industry advancement.

*Pyropia haitanensis* is an endemic warm-temperate macroalga native to China. In 2024, its aquaculture area totaled approximately 6.39 × 10^4^ hectares, with an annual dry weight yield of approximately 2.089 × 10^5^ tons (Fishery administration of Ministry of Agriculture and Rural Affairs, 2025). Currently, the five existing varieties of *P. haitanensis* are pure lines cultivated for commercial application by transplanting free-living filaments into shells. However, the conchocelis stage of current *P. haitanensis* cultivars consistently display delayed maturation and arrested developmental—physiological constraints that critically limit large-scale seedling production and industrial adoption. Therefore, elucidating the molecular mechanisms of underlying *P. haitanensis* conchocelis development is crucial for its precise regulation.

Research on *Porphyra* conchocelis development typically centers on key physiological indicators and gene expression. For example, studies have shown that phycobiliprotein (PBP) content increases with conchocelis maturity in *Pyropia dentata*, thereby enhancing light-harvesting efficiency in conchosporangial branches and promoting both the light-dependent reactions of photosynthesis and carbohydrate biosynthesis (Zhong et al., 2016). Our previous study (Lin et al., 2021) proposed that DGK-mediated phosphatidic acid production activates downstream effectors to maintain membrane and cell wall integrity, stimulate biosynthesis of alkaloids, sugars, and organic metabolites, enhance conchosporangial branches stress resilience, and facilitate energy accumulation for conchospore release. However, the mechanistic basis by which *P. haitanensis* conchocelis transduces maturation-inducing environmental signals into downstream developmental pathways remains unresolved.

Recent research has identified hydrogen peroxide (H_2_O_2_) as a pivotal signaling molecule governing the transition from conchocelis to conchosporangial branches in *P. haitanensis* (Niu et al., 2024). Polyamine spermine promotes conchosporangial branches formation in *P. haitanensis* through signaling regulation involving superoxide anions (O_2_^-^) (Niu et al., 2025). In *Pyropia yezoensis*, H_2_O_2_ induces the transformation of vegetative cells into monospores, thereby facilitating gametophyte production through asexual reproduction (Takahashi and Mikami, 2017). Additionally, during fertilization and post-fertilization development in the red alga *Bostrychia moritziana*, ROS are instantly generated as H_2_O_2_ upon sperm attachment to female trichogynes, ultimately influencing sperm mitosis, gamete fusion, and zygote development (Shim et al., 2022). These findings highlight the significance of investigating how H_2_O_2_ regulates the maturation of free filaments in *P. haitanensis*.

In summary, this study applied the H_2_O_2_ scavenger dimethylthiourea (DMTU) during the conchocelis maturation phase of *P. haitanensis* and systematically elucidated the regulatory role of H_2_O_2_ in conchocelis development through integrated physiological, biochemical, transcriptomic, and metabolomic analyses. Multi-omics integration identified key regulatory genes and differentially accumulated metabolites, thereby revealing H_2_O_2_-mediated pathways governing conchocelis maturation. These results are significant for revealing developmental patterns of alternation of generations in red algae and guiding efficient seedling cultivation and quality improvement in the *P. haitanensis* industry.

## 2 Materials and methods

### 2.1 Experimental materials and reagents

Experimental materials: The free-living conchocelis strain WO84–1 was obtained from the *P. haitanensis* Germplasm in Fujian Province.

Experimental reagents: 1,3-Dimethylthiourea (DMTU; Shanghai Yuanye Bio-Technology Co., Ltd., Shanghai, China; Cat. No. S71061) was used as an H_2_O_2_ scavenger.

Normal conchocelis culture medium: Natural seawater with 1.72 mg/L P (NaH_2_PO_4_·2H_2_O)and 7.78 mg/L N (NaNO_3_) added, and the seawater was disinfected. Conchocelis culture medium with DMTU added: Natural seawater with 1.72 mg/L P (NaH_2_PO_4_·2H_2_O) and 7.78 mg/L N (NaNO_3_) added, and DMTU was added to prepare a 500 μmol·L^-1^ solution and the seawater was disinfected. This concentration was selected based on previous pharmacological studies in which 500 μmol L^-^¹ DMTU was effectively used to suppress H_2_O_2_-dependent signaling in developmental processes (Cui et al., 2017; Zhang et al., 2019).

Conditions for promoting maturation of free-living conchocelis of *P. haitanensis*: (1) Water temperature: 28.5 ± 0.5°C; (2) Light intensity: 20-30 μmol·m^-2^·s^-1^; (3) Photoperiod: Light time: Dark time = 9 h: 15 h; (4) Replace the 28.5°C culture medium every 7 days.

### 2.2 Determination of maturity, daily growth rate and chlorophyll fluorescence parameters Fv/Fm of free-living conchocelis forms of P. haitanensis

The maturity of the free conchocelis of *P. haitanensis* was assessed every 7 days. After the conchocelis forms were shaken evenly, random samples were taken and separated using a dissecting needle. The samples were then prepared on slides and observed under a light microscope at 400× magnification. The number of conchosporangial branches and conchocelis was counted in each field of view.

The maturity index for conchosporangia was calculated as follows:

Maturity (%) = (Number of conchosporangial branches/Total number of conchocelis filaments) × 100% (Lin et al., 2021).

After 28 days of cultivation under either normal maturation-promoting conditions or DMTU treatment, the fresh weight (FW) of free-living *P. haitanensis* conchocelis was measured. The daily fresh weight growth rate (FW-DGR) was calculated as:FW-DGR (mg·day^-^¹) = (FW_28_ − FW_0_)/28, where FW_0_ and FW_28_ denote the fresh weights (mg) measured on day 0 and day 28, respectively.

Fv/Fm, as an important indicator of the maximum photochemical quantum efficiency of the photosystem II, in this study, the fluorescence instrument DIVING-PAM (DivingPAM, Walz, Germany) was used to measure the photosynthetic parameters of the free conchocelis of *P. haitanensis* during the maturation process at different time points. After the fresh algal samples were dark-adapted for 15 minutes, the instrument was used to fix the algae with the leaf clamp. F_0_ represents the fluorescence baseline value when all reaction centers of the photosystem II are in an open state, and Fm represents the fluorescence peak when they are closed. According to the formula Fv/Fm = (Fm - F_0_)/Fm, Fv/Fm was calculated.

### 2.3 RNA extraction, transcriptome sequencing and qRT-PCR

Different treatment conditions and maturation time points were selected as experimental factors. A total of 12 sampling groups were established, as follows:

- Non-maturation control group: 0-day treatment;
- Control and DMTU-treated groups: four time points (7, 14, 21, and 28 days), with both a control and a DMTU-treated subgroup at each time point, yielding 8 groups in total;
- DMTU-treatment-and-recovery groups: DMTU-39d (continuous DMTU treatment for 39 days); DMTU-14d-R25d (14 days of DMTU treatment followed by 25 days of normal maturation-inducing conditions); and DMTU-28d-R11d (28 days of DMTU treatment followed by 11 days of normal maturation-inducing conditions); totaling 3 groups.

Each sample (0.1 g fresh weight) was ground in liquid nitrogen, and total RNA was extracted from *P. haitanensis* conchocelis using the R6827 RNA Extraction Kit (Omega Bio-Tek, USA), according to the manufacturer’s instructions. Total RNA purity (OD_260_/_280_ and OD_260_/_230_ ratios) was assessed using a NanoPhotometer spectrophotometer (Implen). RNA concentration was accurately quantified with a Qubit 2.0 Fluorometer, and RNA integrity was verified using an Agilent 2100 bioanalyzer. For library construction, 2 μg of high-quality total RNA per sample was used as input material, resulting in 36 RNA-Seq libraries. RNA sequencing and bioinformatic analysis were performed on the Illumina platform by Genedenovo Biotechnology Co., Ltd (Guangzhou, China). Raw reads were subjected to quality control using fastp v0.18.0 with the parameters -q 20 -u 50 -N 15 -l 50. The clean reads were aligned to the P. haitanensis reference genome using HISAT2 v2.1.0 with default parameters. Transcript reconstruction was performed using StringTie v1.3.4 with the parameter -f 0.3, and gene expression levels were quantified using RSEM v1.2.19. Differential expression analysis was performed using DESeq2 based on read count data. Genes with an FDR < 0.05 and |log_2_(fold change)| > 1 were considered differentially expressed genes (DEGs). KEGG enrichment analyses of DEGs was performed using the KEGG database. WGCNA was performed using the default settings of the Omicsmart platform.

To validate the reliability of the transcriptome data, nine differentially expressed genes were randomly selected for qRT-PCR analysis (**Supplementary Table 1**). *P. haitanensis* ubiquitin-conjugating enzyme (UBC) was used as the reference gene for normalization of gene expression during free-living conchocelis maturation. The qRT-PCR reaction mixture (20 μL total volume) consisted of: 10 μL SYBR Green Master Mix (AG, China), 6.8 μL nuclease-free water, 2 μL diluted cDNA template (1:10), 0.4 μL forward primer (20 μmol·L^-^¹), 0.4 μL reverse primer (20 μmol·L^-^¹), and 0.4 μL ROX Reference Dye. Thermal cycling conditions were: initial denaturation at 95 °C for 30 s; followed by 40 cycles of 95 °C for 5 s and 60 °C for 30 s. As shown in **Supplementary Figure 1**, the expression trends of the nine genes were highly consistent with those obtained from RNA-Seq. These results confirm the high accuracy and reliability of the RNA-Seq data generated in this study.

### 2.4 Broadly targeted metabolomics analysis

A total of 30 samples were selected and divided into 10 groups for metabolic profiling. Each sample was ground into a fine powder using a grinding mill (MM 400, Retsch) at 30 Hz for 1.5 minutes. Then, 30 mg of the powdered sample was mixed with 1500 μL of pre-cooled (−20 °C) 70% methanol aqueous solution containing internal standards. The mixture was vortexed every 30 seconds for a duration of 30 seconds, repeated six times. After centrifugation at 12,000 rpm for 3 minutes, the supernatant was collected and filtered through a microporous membrane (0.22 μm pore size) prior to UPLC-MS/MS analysis. Mass spectrometry data were processed using Analyst 1.6.3 software. Metabolites were identified based on secondary spectral information using the Metware database (MWDB). During analysis, isotopic signals, as well as duplicate signals from fragments such as K^+^, Na^+^, NH_4_^+^, or those originating from larger molecular weight compounds, were excluded. Quantification of metabolites was performed using multiple reaction monitoring (MRM) mode on a triple quadrupole mass spectrometer. Differentially accumulated metabolites were identified based on VIP > 1 and fold change (FC) ≥ 2 or FC ≤ 0.5.

### 2.5 Statistical analysis

Each experiment included three biological replicates per group, and all results are expressed as “mean ± standard deviation (SD)”. Data were statistically analyzed using SPSS version 24.0 (SPSS Inc., Chicago, IL, USA). One-way analysis of variance (one-way ANOVA) was first applied to assess significant differences among treatment groups, followed by Tukey’s Honestly Significant Difference (Tukey’s HSD) test for *post-hoc* multiple comparisons. A P-value < 0.05 was considered statistically significant.

## 3 Results

### 3.1 The effect of DMTU treatment on conchocelis maturation

During the maturation-inducing process of *P. haitanensis* conchocelis, the morphology and the number of conchosporangial branches were examined microscopically at 7-day intervals. Observations revealed that at 14, 21, and 28 days of maturation, the CK group formed more conchosporangial branches than the DMTU-treated group (**Figure 1A**). At day 39, the maturation progression of the DMTU-14d-R25d group (14 days of DMTU treatment followed by 25 days of normal maturation) and the DMTU-28d-R11d group (28 days of DMTU treatment followed by 11 days of normal maturation) was intermediate between that of the CK and DMTU-treated groups; notably, the upward trend of the DMTU-14d-R25d group more closely approximated that of the CK group (**Figure 1B**).

**Figure 1:**
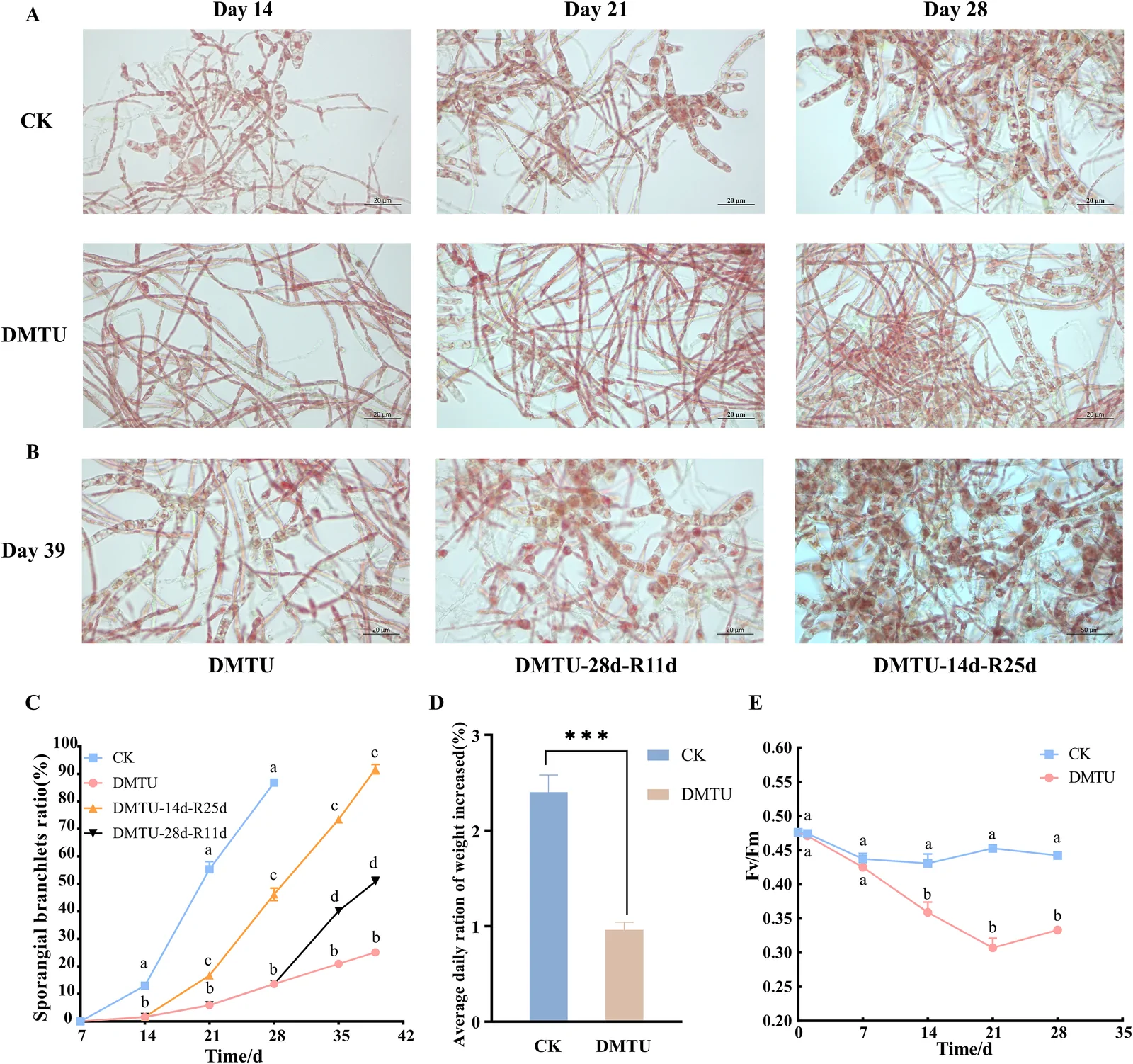
Effects of DMTU treatment on the maturation of free-living conchocelis of (P) haitanensis WO84-1. **(A, B)** Representative micrographs of conchocelis under different maturation-inducing conditions; **(C)** maturity; **(D)** daily fresh-weight growth rate; and **(E)** maximum quantum yield of PSII (Fv/Fm). Data are presented as mean ± SD of three biological replicates (n = 3). Different lowercase letters indicate significant differences among treatment groups at the same time point, as determined by one-way ANOVA followed by Tukey’s HSD test (P < 0.05); identical letters indicate no significant difference. For pairwise comparisons indicated by brackets, *** denotes P < 0.001.

Maturity assessment results (**Figure 1C**) indicate that as maturation duration extended, the rate of maturity increase in the DMTU-treated group progressed slowly and remained consistently lower than that of the CK group. **Figure 1D** further revealed that DMTU treatment significantly inhibited the daily fresh weight growth rate compared with the CK group. The Fv/Fm of the CK group exhibited minor fluctuations throughout the maturation process but remained relatively stable overall. In contrast, the Fv/Fm of the DMTU-treated group showed a trend of first decreasing and then increasing, reaching the lowest value at 21 days, and from 14 days after the maturation began, the Fv/Fm of the DMTU-treated group was significantly lower than that of the CK group (**Figure 1E**). Collectively, these results demonstrate that DMTU significantly inhibit the formation of conchosporangial branches of *P. haitanensis*, and this inhibitory effect is reversible.

### 3.2 Changes in ROS content and antioxidant system in free-living conchocelis of under DMTU treatment

During the maturation-inducing process, H_2_O_2_ content in the CK group fluctuated, whereas it remained significantly lower than in the CK group at all time points. The O_2_^-^ content in the CK group initially increased and then declined, while it remained consistently low in the DMTU-treated group, with levels being significantly higher in the CK group than in the DMTU-treated group. The ·OH scavenging rate gradually declined in the CK group but showed a trend of first decreasing and then increasing in the DMTU-treated group. Notably, at 21 days and 28 days, the ·OH scavenging rate was significantly higher in the DMTU-treated group than in the CK group (**Figure 2**). Antioxidant enzyme activities (**Supplementary Figures 2A–C**) revealed that the SOD activity in the CK group showed a trend of first increasing and then decreasing, reaching its peak at 21 days. Overall, SOD activity in the DMTU-treated group was higher than that in the CK group. CAT activity in the CK group first decreased and then increased, reaching its peak at 14 days. The DMTU-treated group exhibited a similar trend but lower activity than the CK group. The GR activity in the CK group continued to decline, while in the DMTU-treated group, it first decreased and then increased. Only at 7 days of maturation was GR activity in the DMTU-treated group significantly higher than that in the CK group.

**Figure 2:**
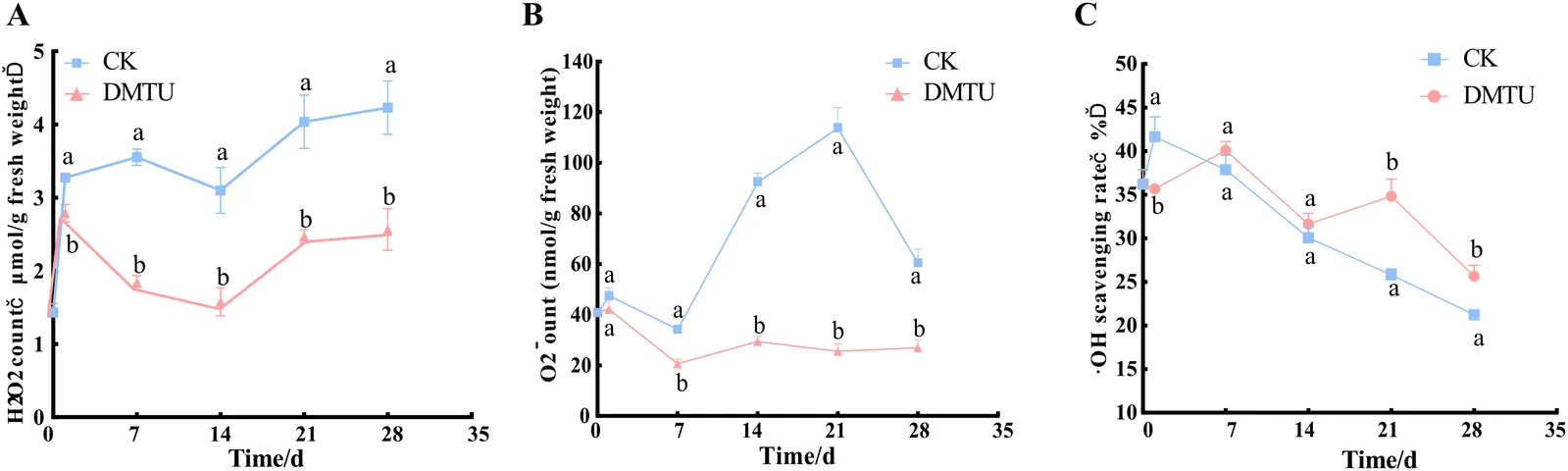
Effects of DMTU treatment on ROS levels in free-living conchocelis of (P) haitanensis. **(A)** H_2_O_2_ content; **(B)** O_2_^-^ content; and **(C)** OH scavenging rate. Data are presented as mean ± SD of three biological replicates (n = 3). Different lowercase letters indicate significant differences among treatment groups at the same time point, as determined by one-way ANOVA followed by Tukey’s HSD test (P < 0.05); identical letters indicate no significant difference.

### 3.3 Transcriptome analysis of free-living conchocelis in *P. haitanensis* under DMTU treatment.

In this study, 12 groups of free-living conchocelis in *P. haitanensis*, sampled at different time points during maturation induction under distinct treatment conditions, were subjected to transcriptome analysis. Principal component analysis (PCA) revealed that the three biological replicates within each group clustered tightly with low inter-replicate variation, while the CK and DMTU-treated groups formed distinct, separate clusters (**Supplementary Figure 3A**). Pearson correlation coefficient analysis further confirmed high consistency in gene expression profiles among biological replicates within each group (**Supplementary Figure 3B**).

Differential gene expression was compared between the CK and DMTU-treated groups across eight sample groups at various maturation-inducing time points, with the number of differentially expressed genes (DEGs) summarized in **Figure 3A**. Across the eight sample groups, 8,374 genes were detected in total, of which 1,694 were identified as DEGs. At day 7 of maturation induction, the number of DEGs between the CK group and the DMTU treated groups reached its peak at 848 (**Figure 3A**). At day 39, 1,296 DEGs were identified between the DMTU-treated and DMTU-14d-R25d groups, while 673 DEGs were detected between the DMTU-treated and DMTU-28d-R11d groups (**Figure 3B**).

**Figure 3:**
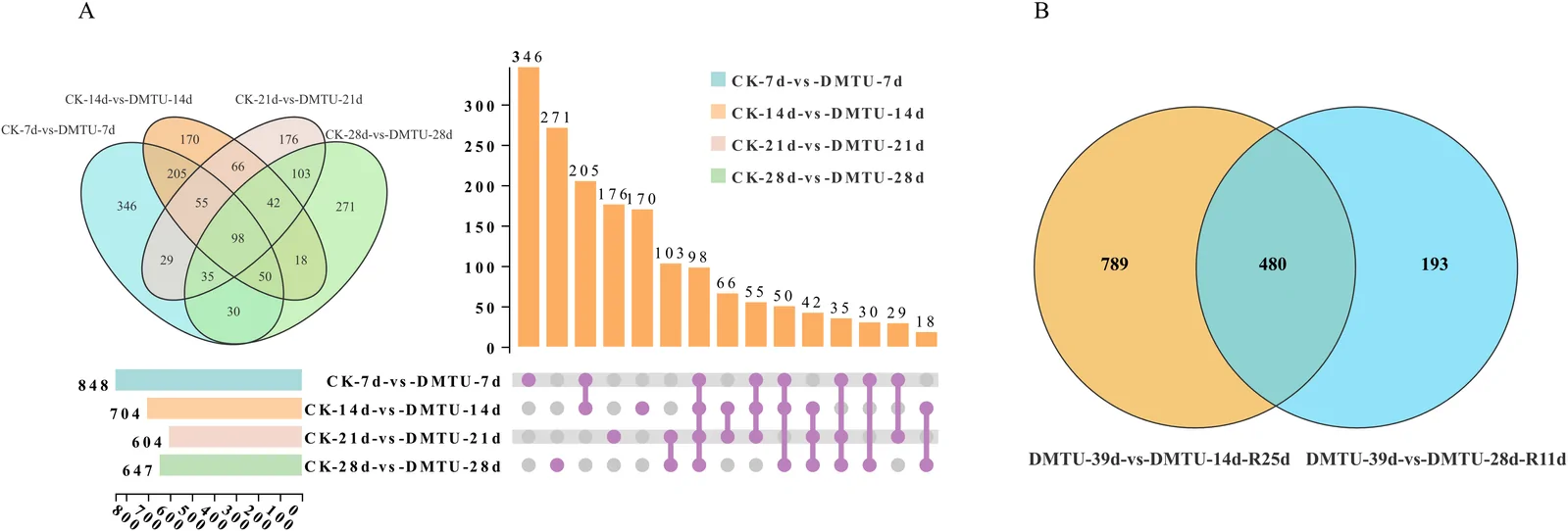
UpSet and Venn analyses of differentially expressed genes (DEGs). **(A)** UpSet plot showing the distribution of DEGs among different comparisons; **(B)** Venn diagram showing DEGs among treatment and recovery groups at day 39.

KEGG enrichment analysis of DEGs (**Supplementary Figure 4**) revealed that at day 7 of maturation induction, DEGs between treatment groups were primarily enriched in pathways including the peroxisome pathway, plant MAPK signaling pathway, and photosynthesis—antenna proteins. At day 14, DEGs were predominantly enriched in sulfur metabolism, nitrogen metabolism, carbon metabolism, and the plant MAPK signaling pathway. At day 21, DEGs were predominantly enriched in sulfur and nitrogen metabolism pathways. At day 28, DEGs were significantly enriched in nitrogen metabolism, carbon metabolism, and sulfur metabolism pathway. When the DMTU-treated group was used as the control, DEGs from both the DMTU-14d-R25d and DMTU-28d-R11d groups were consistently enriched in key metabolic pathways, including sulfur metabolism, nitrogen metabolism, photosynthesis, glycine–serine–threonine metabolism, and α-linolenic acid metabolism.

Heatmap analysis of DEGs further revealed that DMTU treatment consistently suppressed genes associated with photosynthesis and nitrogen metabolism across all maturation time points. Upon restoration of normal maturation conditions, certain genes involved in photosynthesis and nitrogen metabolism were upregulated relative to those maintained under continuous DMTU treatment. Genes associated with carbon metabolism and antioxidant systems exhibited transcriptional reallocation. Concurrently, a subset of MAPK signaling-related genes was downregulated during maturation, whereas genes associated with the phosphatidylinositol (PI) signaling system, heat shock proteins, and sulfur metabolism were upregulated (**Figure 4**).

**Figure 4:**
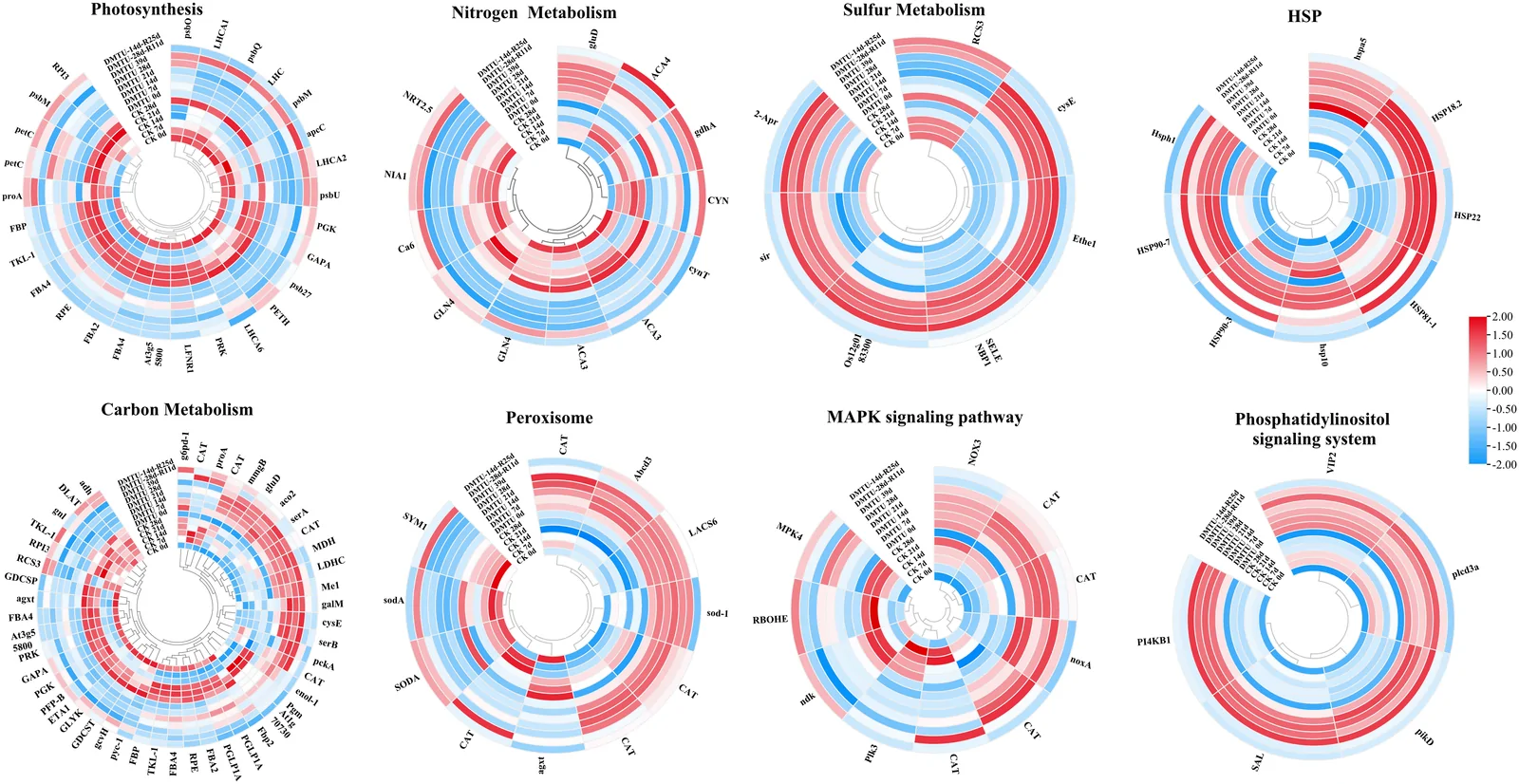
Expression patterns of selected differentially expressed genes associated with major functional pathways under different maturation-inducing conditions. Genes are grouped according to their functional annotations, including photosynthesis, nitrogen metabolism, sulfur metabolism, peroxisome, carbon metabolism, the MAPK signaling pathway, heat shock proteins (HSPs), and phosphatidylinositol (PI) signaling. Identical gene symbols denote distinct gene models with the same functional annotation. Colors indicate relative gene expression levels, with red and blue representing higher and lower expression, respectively.

All genes obtained from RNA-seq were subjected to Weighted Gene Co-expression Network Analysis (WGCNA). By comparing the expression trends relative to the CK group, three modules of interest were identified: thistle1, bisque4, and cyan. In the thistle1 module, genes in the CK group exhibited high expression at days 7 and 14 of maturation induction, while the corresponding genes in the DMTU-treated group remained at low expression levels. In the cyan module, genes in the CK group exhibited elevated expression at days 21 and 28 of maturation induction, but no corresponding upregulation was observed in the DMTU-treated group during the same period. In the bisque4 module, genes in the CK group maintained consistently low expression levels throughout the entire maturation process, whereas those in the DMTU-treated group exhibited persistently high expression.

Key genes identified from the gene co-expression network during the early stage of maturation induction (days 7 and 14) included DNA repair and recombination proteins (RAD54B and RAD51), DNA-3-methyladenine glycosylase (Tag), DNA damage-binding protein 2 (DDB2), flap endonuclease 1 (FEN1), 26S proteasome regulatory subunit N9 (PsmD13), ribonucleotide diphosphate reductase subunit M1 (RNR1), and mitogen-activated protein kinase MAPK7/14 (ErkA) (**Figure 5D**). Key genes during the late stage of maturation induction (days 21 and 28) included ribulose phosphate 3-epimerase (RPE), 1,4-α-glucan branching enzyme (GLC3), ferredoxin-NADP^+^ reductase (LNFR1), thioredoxin reductase (NTRC), fructose-1,6-bisphosphate aldolase (FBA2 and FBA4), phosphoketolase (At1g32060), and transketolase (TKL-1) (**Figure 5E**). During continuous DMTU treatment, key genes that were persistently upregulated throughout the maturation process included serine O-acetyltransferase (CysE), methionol oxidase (SELENBP1), ATP-binding cassette transporters (ABCB6 and ABCG27), heat shock protein 22 (HSP22), phosphatidylinositol 3-kinase (PikD), and phosphoserine phosphatase (Sco1808) (**Figure 5F**).

**Figure 5:**
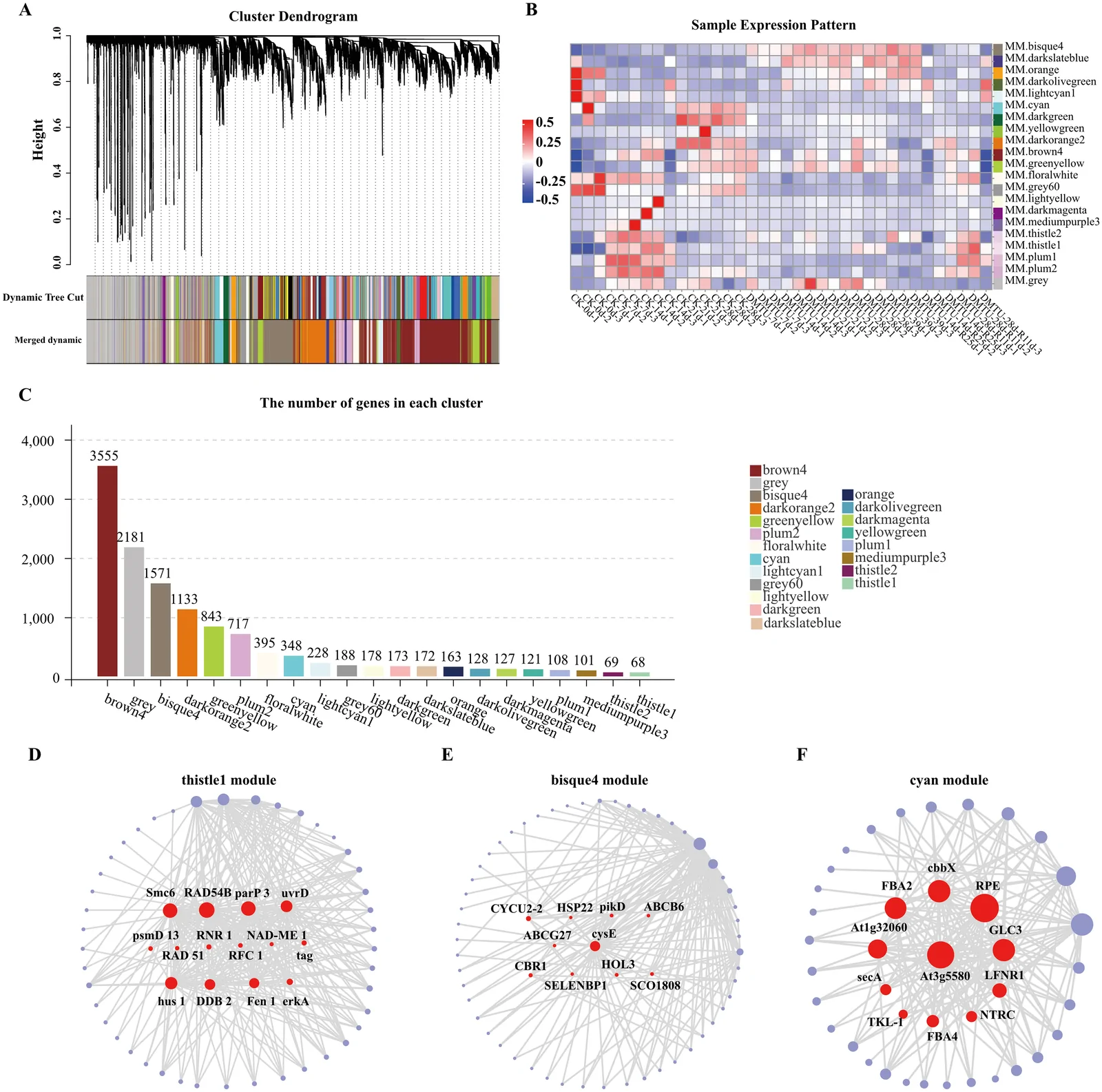
Weighted gene co-expression network analysis (WGCNA) of transcriptomic data. **(A)** Gene clustering dendrogram; **(B)** expression patterns of selected modules; **(C)** number of genes in each module; and **(D–F)** co-expression networks of key genes in selected modules.

### 3.4 Metabolomic analysis of free-living conchocelis in *P. haitanensis* under DMTU treatment.

In this study, 30 samples were divided into 10 groups for metabolomic analysis. In total, 1,448 metabolites were identified, with amino acids and their derivatives constituting the most abundant category (22.44%), followed by lipids (18.99%). PCA performed on all identified metabolites revealed a clear separation trend among groups, enabling effective discrimination of metabolic differences between sample groups. Notably, the CK group was clearly separated from the DMTU-treated group, whereas the recovery culture groups clustered within the same quadrant as the CK-21d and CK-28d groups (**Figure 6B**). Further screening of differential metabolites (VIP > 1 and FC ≥ 2 or FC ≤ 0.5) combined with Venn diagram analysis identified 64 common differential metabolites across three maturation time points (days 14, 21, and 28), based on pairwise comparisons of DMTU-14d vs. CK-14d, DMTU-21d vs. CK-21d, and DMTU-28d vs. CK-28d (**Figure 6C**). At all three time points, down-regulated metabolites substantially outnumbered up-regulated metabolites. At day 39 of maturation induction, 134 shared differential metabolites were identified between the DMTU-14d-R25d vs. DMTU-39d and DMTU-28d-R11d vs. DMTU-39d comparisons (**Figure 6D**). Statistical analysis revealed that differential metabolites numbered 187 at day 14 (59 up-regulated, 128 down-regulated), 275 at day 21 (63 up-regulated, 212 down-regulated), and 221 at day 28 (98 up-regulated, 123 down-regulated). At all three time points, down-regulated metabolites consistently outnumbered up-regulated metabolites. Comparisons of DMTU-14d-R25d vs. DMTU-39d and DMTU-28d-R11d vs. DMTU-39d revealed 256 and 201 differential metabolites, respectively, with up- and down-regulated metabolites showing a relatively balanced distribution (**Figure 6E**).

**Figure 6:**
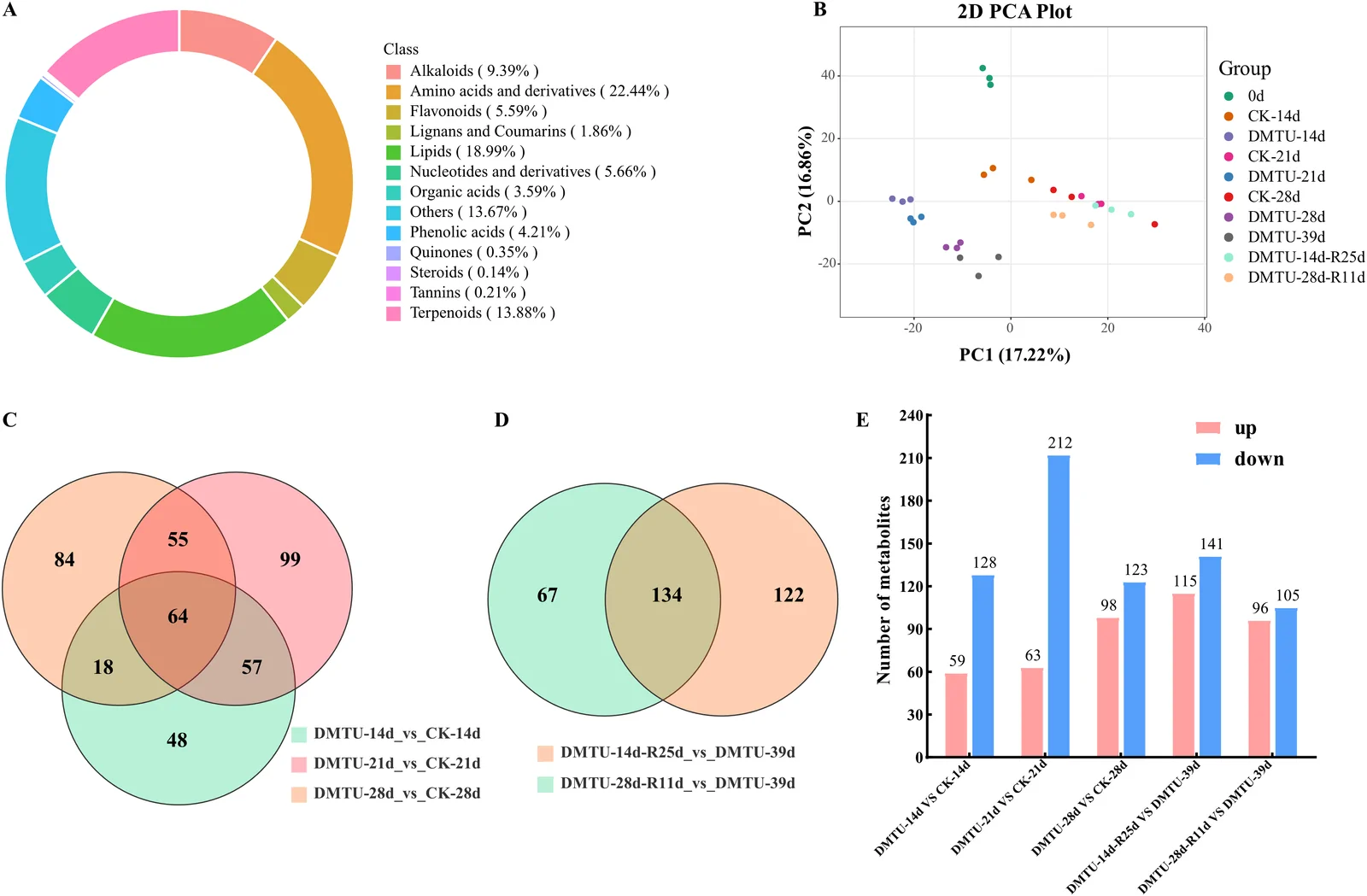
Metabolomic profiles and differential metabolite analysis under different maturation-inducing conditions. **(A)** Classification of identified metabolites; **(B)** principal component analysis (PCA) of metabolite profiles; **(C)** Venn analysis of differential metabolites between CK and DMTU-treated groups at days 14, 21, and 28; **(D)** Venn analysis of differential metabolites between the continuous DMTU treatment and recovery groups at day 39; and **(E)** numbers of up- and down-regulated differential metabolites in the indicated comparisons. Each treatment group contained three biological replicates (n = 3).

The enriched pathways of differential metabolites displayed pronounced time-dependent variations as the maturation induction period progressed. At days 14, 21, and 28, differential metabolites between the CK and DMTU-treated groups were predominantly enriched in purine metabolism, nucleotide metabolism, lipid metabolism, and alanine–aspartate–glutamate metabolism. At day 39 of maturation induction, differential metabolites in both the DMTU-14d-R25d and DMTU-28d-R11d groups, relative to the DMTU-treated group, were enriched in zeatin biosynthesis, flavonoid biosynthesis, purine metabolism, the tricarboxylic acid (TCA) cycle, alanine–aspartate–glutamate metabolism, nucleotide metabolism, and ether lipid metabolism (**Supplementary Figure 5**).

To further elucidate the regulatory effects of DMTU treatment on conchocelis maturation in *P haitanensis*, we focused on three KEGG-enriched pathways: lipid metabolism, nucleotide metabolism, and flavonoid biosynthesis. A total of 105 differentially expressed lipids metabolites were identified (**Supplementary Figure 6A**). At days 14, 21, and 28 of maturation promotion, LPC and LPE levels in the DMTU-treated group were significantly lower than those in the CK group, but upon restoration of normal maturation conditions, these levels were significantly higher in the DMTU-treated group than in the CK group. A total of 52 differentially expressed nucleotide metabolites were identified (**Supplementary Figure 6B**). At days 14, 21, and 28 of maturation promotion, the levels of purine metabolites, pyrimidines, and other nucleotide metabolites in the DMTU-treated group were significantly lower than those in the CK group. At day 39, the levels of certain nucleotide metabolites in the DMTU-14d-R25d and DMTU-28d-R11d groups were significantly higher than those in the DMTU-39d group, whereas the levels of a few nucleotide metabolites were lower. A total of 48 differentially accumulated flavonoid metabolites were identified. The majority of these flavonoid metabolites exhibited significantly differential accumulation between the DMTU-treated and CK groups. Throughout the entire maturation process, the level of quercetin-3-O-sambubioside-5-O-glucoside was consistently lower in the DMTU-treated group than in the CK group. At day 39 of maturation promotion, most flavonoid compounds showed significantly reduced levels in both recovery groups—DMTU-14d-R25d and DMTU-28d-R11d—compared to the continuously treated DMTU-39d group (**Supplementary Figure 6C**).

### 3.5 Integrated transcriptomic and metabolomic analysis

Based on loading values, the top 10 genes and metabolites with the highest sum of squared loadings across two dimensions were selected for integrated analysis and visualized in a loading plot (**Figure 7A**). The metabolites most strongly associated with the transcriptome included amino acids and their derivatives, such as L-arginyl-L-prolyl-L-asparagine (MW0145476), 4-methylene-L-glutamine (Zkep143399), glutaminyl-glutaminyl-alanine (MW0149661), L-proline (MWStz040), and N-acetylaspartylglutamate (MEDN0561); lipids, such as lysophosphatidylcholine 17:1 (Lmhp009590), phosphatidylcholine lysoform 17:1 (PDP239492), lysophosphatidylglycerol 16:0 (Hmyn007168), and arachidonic acid (MEDL00447); and terpenoids, such as hispanolone (pmp000970).Furthermore, correlation analysis was conducted using quantitative values of genes and metabolites across all samples. DEGs and DMs with an absolute Pearson correlation coefficient greater > 0.8 and p < 0.05 were selected; following the removal of unannotated DEGs, a correlation network was constructed, comprising 22 key metabolites (**Figure 7B**). These 22 key metabolites comprised 12 amino acids and their derivatives, 6 lipids, 2 alkaloids, and 2 terpenoids. At each maturation induction time point, the accumulation trends of these key metabolites in the DMTU-treated group were inversely correlated with those in the CK group. Upon restoration of normal maturation conditions, the accumulation trends of these metabolites progressively converged with those of the CK group, with longer recovery periods associated with greater consistency (**Figure 7C**).

**Figure 7:**
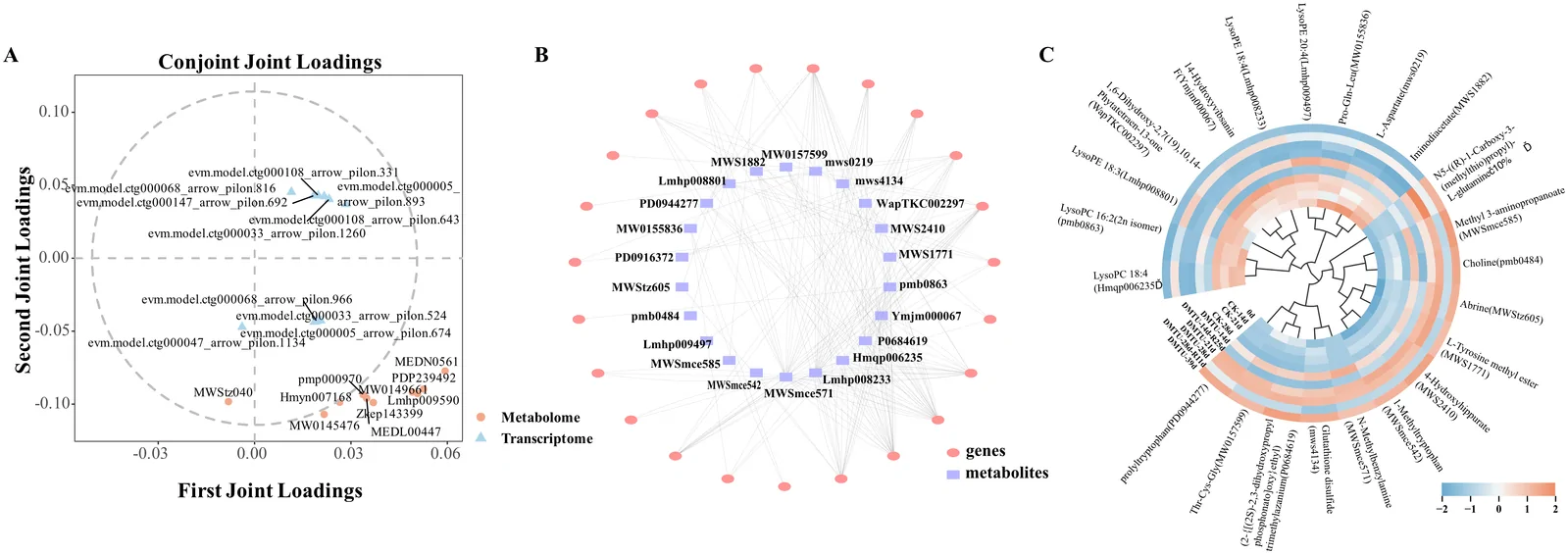
Integrated transcriptomic and metabolomic analysis. **(A)** Loading plot of genes and metabolites; **(B)** correlation network of differentially expressed genes and differential metabolites; and **(C)** heatmap showing the accumulation patterns of key metabolites under different maturation-inducing conditions.

## 4 Discussion

### 4.1 The effect of H_2_O_2_ on the maturation of free-living conchocelis of *P. haitanensis*

Continuous DMTU application during the conchocelis maturation phase significantly suppressed vegetative growth and developmental progression in free-living P. haitanensis conchocelis, as evidenced by a substantially reduced Fv/Fm ratio compared to controls. However, conchosporangial branch formation fully recovered after DMTU-treated conchocelis were returned to normal maturation-inducing conditions, and even after a longer period of DMTU treatment, maturity increased rapidly once normal conditions were restored, indicating that the developmental arrest was reversible rather than resulting from irreversible physiological impairment. Thus, extending previous evidence for an early signaling role of H_2_O_2_, our study shows that sustained H_2_O_2_ depletion delays conchocelis maturation and further demonstrates that this developmental inhibition is largely reversible. This reversibility provides a practical advantage: DMTU treatment can delay maturation induction under unfavorable conditions and effectively reduce conchospore “abortion.”

DMTU inhibits conchocelis maturation primarily via H2O2 scavenging, evidenced by two key observations: first, it significantly reduces H_2_O_2_ levels and is accompanied by a decrease in O_2_^-^ levels, the latter of which has been implicated in regulating maturation in free-living conchocelis (Niu et al., 2025); second, it markedly alters the activity of antioxidant enzymes—including SOD and CAT—and modulates the levels of antioxidant metabolites. These observations are consistent with previous findings demonstrating that H_2_O_2_ depletion can disrupt enzymatic activities (Lee et al., 2022; Liu et al., 2015). For instance, in tomato plants, DMTU treatment alleviated cold-induced H_2_O_2_ overaccumulation and concurrently suppressed the upregulation of antioxidant enzymes—including SOD, CAT, and APX—typically induced by cold stress (Zhou et al., 2012). Collectively, direct depletion of H_2_O_2_ by DMTU was accompanied by secondary changes in O_2_^-^ accumulation and antioxidant enzyme activities, indicating a broader disturbance of cellular ROS homeostasis. Together, these changes may disrupt cellular redox balance, interfere with H_2_O_2_-mediated signaling, and ultimately contribute to the suppression of developmental progression in P. haitanensis filaments.

### 4.2 The effect of H_2_O_2_ on photosynthesis and sulfur metabolism of free-living conchocelis in *P. haitanensis*

Under sufficient light, plants efficiently perform carbon assimilation, promoting leaf expansion, stem elongation, and root proliferation, thereby conferring overall growth advantages (Tang et al., 2022; Wu et al., 2025). For example, seed priming with an optimal concentration of H_2_O_2_ enhances the photosynthetic efficiency of *Bombax ceiba* L. seedlings under heat stress (Zheng et al., 2018). In the present study, transcriptome analysis revealed that DMTU treatment suppressed photosynthesis in free-living conchocelis of *P. haitanensis*, accompanied by downregulation of light reaction-related genes. These included genes encoding components of photosystem I (LHCA1, LHCA2, LHCA6) and photosystem II (psbU, psbM, psbQ, psbO), as well as genes involved in electron transport (e.g., petC, LFNR1, PETH, atpF) and the Calvin cycle (GAPA, PGK, PRK) (**Figure 4**). DMTU also affected phycobilisome-related gene expression (**Supplementary Figure 8**). Photosynthetic activity provides the energy required for conchosporangial branches formation; however, DMTU-induced reduction in photosynthetic efficiency impairs the provision of essential energy and protective mechanisms, ultimately disrupting the basic metabolism of the alga.

Photosynthesis and sulfur metabolism are intimately linked. Photosynthesis provides the ATP and NADPH required for sulfur assimilation, while sulfur is an essential constituent of cysteine, methionine, and key vitamins (Takahashi et al., 2010). Sulfate is either sequestered in vacuoles via SULTR4;1 or transported to chloroplasts through SULTR3;1 to initiate assimilation (Gotor et al., 2015; Takahashi et al., 2010). Within chloroplasts, sulfate is activated to 5’-adenylyl sulfate (APS) by ATP sulfurylase (ATPS). APS is subsequently reduced to sulfite by APS reductase (APR), with glutathione (GSH) acting as the reducing agent (Birke et al., 2015). Sulfite is further reduced by sulfite reductase (SiR) to sulfide (Fu et al., 2018), which serves as the sulfur donor for cysteine biosynthesis. Cysteine biosynthesis proceeds through two sequential reactions: first, serine acetyltransferase (SAT) catalyzes the condensation of acetyl-CoA with serine to form O-acetylserine (OAS), second, OAS is conjugated with sulfide by cysteine synthase (CysK) to produce cysteine (Álvarez et al., 2010).

Transcriptome analysis of sulfur metabolism genes revealed significant upregulation of SiR, methanethiol oxidase (SELENBP1), sulfur dioxygenase (Ethe1), adenosine phosphosulfate reductase (APR), and serine O-acetyltransferase (cysE) in the DMTU-treated group compared to the CK group, with only cysteine synthase (CysK) being significantly downregulated (**Figure 4**). Under normal maturation conditions, algae likely enhance cysteine synthesis to support protein synthesis and conchosporangial branches development. However, DMTU treatment inhibits photosynthesis and nitrogen metabolism, prompting algae to upregulate sulfur metabolism as a stress response. This adaptive adjustment limits synthesis of cysteine required for growth, ultimately delaying the maturation of conchocelis.

### 4.3 The effect of H_2_O_2_ on signal transduction pathways during maturation of free-living conchocelis in *P. haitanensis*

H_2_O_2_ functions both as a metabolic byproduct and as a crucial signaling molecule that regulates growth, development, and stress responses. H_2_O_2_-mediated signaling plays diverse roles in plant cells (Mittler et al., 2022; Xiong et al., 2015), including the regulation of stress perception, defense mechanism activation, and fruit maturation (Dumanovic et al., 2020). In this study, DMTU treatment significantly reduced H_2_O_2_ levels, resulting in alterations in both the mitogen-activated protein kinase (MAPK) cascade and the phosphatidylinositol (PI) signaling pathway.

On day 14 of the maturation-induction period, MPK4 expression was significantly downregulated in the DMTU-treated group. However, on day 39, MPK4 expression was significantly upregulated in the DMTU-14d-R25d recovery group compared to the DMTU-treated group (DMTU-39d). The MAPK cascade comprises three sequentially activated tiers—MAPKKK, MAPKK, and MAPK—that undergo phosphorylation-dependent activation. This pathway serves as a central regulatory hub that governs diverse cellular processes, including cell proliferation, differentiation, and stress responses (Pan and De Smet, 2020). Under stress conditions, upstream signals activate the MAPK cascade through multiple coordinated mechanisms: transcriptional reprogramming of stress-responsive genes, induction of antioxidant defenses, and cross-talk among distinct signaling pathways, collectively enhancing stress tolerance (Li et al., 2022). Attenuation of MAPK activity disrupts cell cycle progression and impairs both mitotic and meiotic divisions, thereby compromising vegetative growth and reproductive development (Banerjee et al., 2020). Severe suppression of MAPK signaling abolishes the plant’s capacity to sense and transduce stress signals, resulting in irreversible growth arrest and, ultimately, lethality under adverse conditions (Majeed et al., 2023).

PI signaling pathway—centered on the dynamic turnover and phosphorylation of membrane-associated phosphoinositides—exerts essential regulatory control over plant growth, development, and stress adaptation (Ali et al., 2022). In *Arabidopsis thaliana*, imbalances in LPC and LPE disrupt the elasticity of the endoplasmic reticulum membrane, interfere with vesicle transport, and activate the endoplasmic reticulum stress pathway, ultimately inhibiting root elongation (Wang et al., 2026). In this study reveals that key genes encoding PI signaling pathway components were significantly upregulated (**Supplementary Figure 5**). Concurrently, cellular levels of LPCs and LPEs were markedly reduced compared to the CK group. Upon restoration of normal maturation conditions, LPC and LPE levels recovered (**Supplementary Figure 6A**). These findings suggest that the pronounced decline in algal H_2_O_2_ content attenuates MAPK cascade activation, thereby compromising signal transduction fidelity and impairing growth and development signaling. In response, algae engage a compensatory mechanism involving transcriptional upregulation of the PI signaling pathway. Following restoration of normal maturation conditions, MAPK signaling activity is restored, and LPC/LPE homeostasis recovery coincides with the proper morphogenesis of conchosporangial branches.

### 4.4 The effect of H_2_O_2_ on proteostasis during maturation of free-living conchocelis in *P. haitanensis*

Protein homeostasis is essential for plant growth, development, and stress responses. As central components of the cellular proteostasis network, heat shock proteins (HSPs) function as evolutionarily conserved molecular chaperones that refold misfolded or damaged proteins and prevent cytotoxic protein aggregate accumulation, thereby safeguarding cellular integrity and functional stability under both normal and stress conditions (Khan et al., 2021; Ul Haq et al., 2019). In this study, DMTU treatment induced a general upregulation of genes from the major HSP families (**Figure 4**; **Supplementary Figure 7**), a response that can be mechanistically attributed to two interrelated physiological frameworks: the reinforcement of protein homeostasis and the reallocation of cellular energy resources.

Elevated HSP expression is fundamental to sustaining protein homeostasis and supporting essential physiological processes. For instance, heat stress triggers robust upregulation of HSP70 in the thalli of *P. haitanensis*, a response recognized as a key mechanism for thermal stress tolerance and proteostasis maintenance (Qijun et al., 2014). Similarly, five *PysHSP* genes *Pyropia yezoensis* exhibit consistent induction under high temperature, oxidative stress, and Cu^2+^ exposure, demonstrating their broad-spectrum responsiveness to abiotic stresses and underscoring their functional role in enhancing stress tolerance through proteostatic regulation (Uji et al., 2019).

However, sustained high-level HSP expression imposes a substantial energetic burden on cells. Chloroplast protein import relies on ATP hydrolysis, with HSP70—acting as both a molecular chaperone and an ATP-dependent motor—mediating this process by coupling ATPase activity to conformational changes that drive substrate translocation. Consequently, HSP70 ATPase activity is the primary determinant of energy consumption and efficiency in this pathway (Liu et al., 2014). Given finite cellular energy resources, plants under stress engage in a well-documented physiological trade-off between growth and defense: energy is preferentially redirected toward stress-responsive pathways, including chaperone synthesis and activity, at the expense of growth-related processes such as cell division, expansion, and biomass accumulation (Erofeeva, 2024). DMTU disrupts intracellular ROS homeostasis by scavenging H_2_O_2_, thereby triggering protein damage and misfolding. This proteotoxic stress induces transcriptional and translational upregulation of HSPs to facilitate the refolding or clearance of damaged proteins. To mount an effective defense response, cells prioritizes energy allocation toward stress-responsive pathways—including chaperone synthesis and activity, at the expense of growth-related processes such as cell proliferation, expansion, and biomass accumulation, ultimately suppressing algal growth and development.

## 5 Conclusion

Under normal maturation-inducing conditions, conchocelis exhibited distinct developmental phases. In the early maturation stage, environmental cues—including warming, light reduction, and phosphorus addition—induced H_2_O_2_ production, which promoted rapid nutritional conchocelis growth by enhancing cell proliferation. In the late maturation stage, activated metabolic pathways, such as photosynthesis and carbon metabolism, provided the material and energy basis for conchosporangial branches formation. However, DMTU (500 μmol/L) treatment significantly reduced H_2_O_2_ and O_2_^-^ levels in algae, disrupting ROS homeostasis and altering antioxidant enzyme activities (CAT, SOD). DMTU treatment also inhibited key pathways, including photosynthesis (petc, LHC, Calvin cycle) and nitrogen metabolism (NRT, NR, NiR), and reduced lipid content. Notably, DMTU treatment significantly upregulated the PI signaling pathway and HSP-related gene expression, further enhancing stress response regulation. Particularly in the late maturation stage, algae continuously maintained a stress response state, redirecting energy and resources from the maturation program to the stress resistance program, ultimately inhibiting the transformation of free-living conchocelis into conchosporangial branches (**Figure 8**). Furthermore, the inhibitory effect on conchocelis maturation was reversed and normal maturation was restored when maturation-inducing conditions were resumed after 14 or 28 days of DMTU treatment.

**Figure 8:**
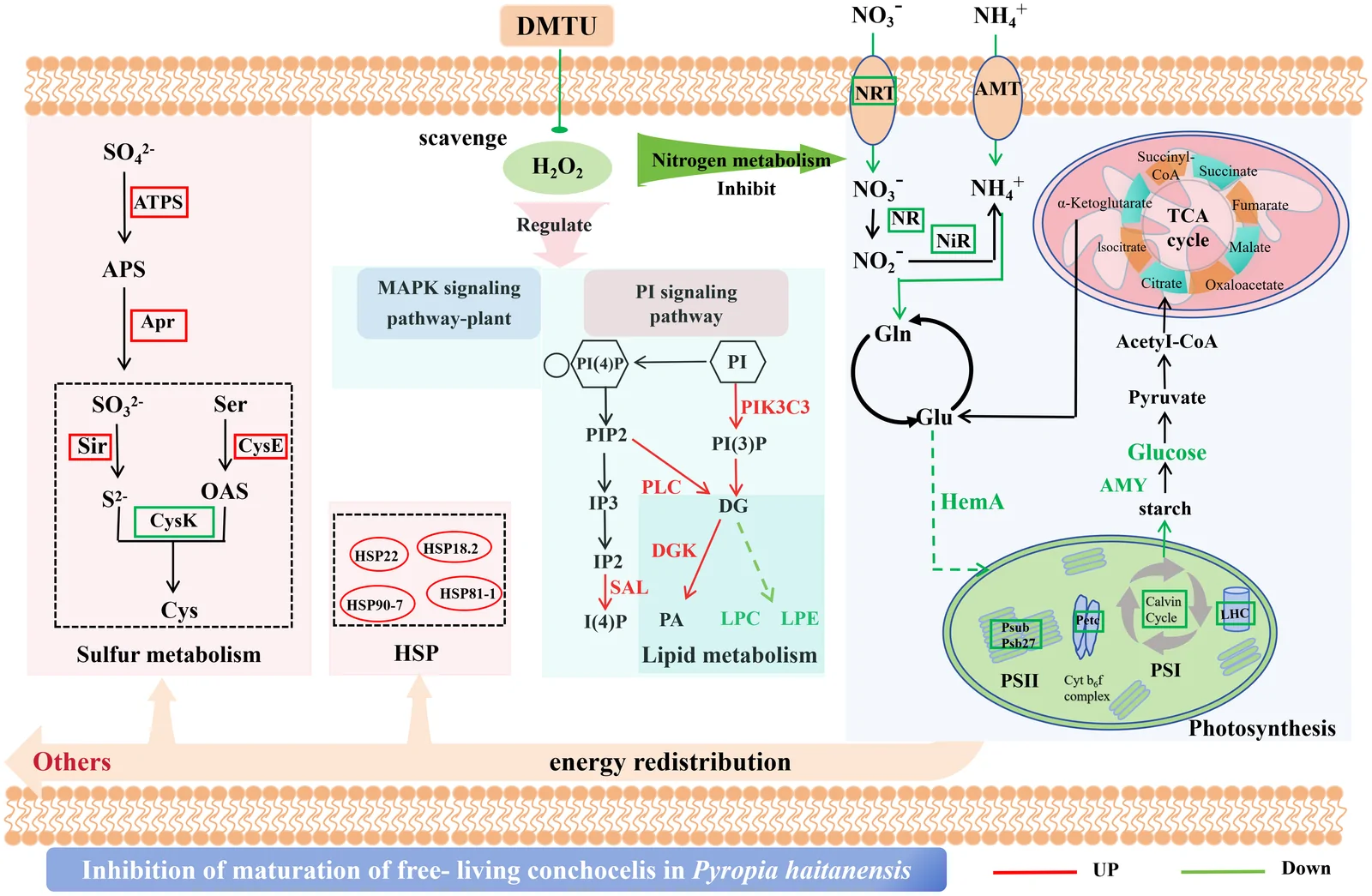
Schematic diagram illustrating the mechanism of H_2_O_2_ regulating the maturation of free-living conchocelis in *Pyropia haitanensis.* The full names of abbreviations used in this figure are listed in **Supplementary Table 2**.

## Data Availability

The original contributions presented in the study are included in the article/**Supplementary Material**. Further inquiries can be directed to the corresponding authors.
