# Supplementary material for: The molecular mechanisms underlying hydrogen peroxide regulating free-living conchocelis maturation of Pyropia haitanensis
Source: Front Plant Sci. 2026 Oct 12;17:1979927. doi: 10.3389/fpls.2026.1979927 (PMC13696270; doi:10.3389/fpls.2026.1979927)
Supplement: Supplementary file 1 [file DataSheet1.pdf]

Supplementary material

The molecular mechanisms underlying hydrogen peroxide regulating free-living conchocelis maturation of *Pyropia haitanensis*

Rong He[1,2,3,#], Wenfei Xian[1,2,3,#], Kai Xu[1,2,3], Yan Xu[1,2,3], Dehua Ji[1,2,3], Chaotian Xie[1,2,3*], Wenlei Wang[1,2,3*].

1. Fisheries College, Jimei University, Xiamen 361021, China

2. State Key.Laboratory of Mariculture Breeding, Fisheries College, Jimei University, Ningde 352100, China

3. Key Laboratory of Healthy Mariculture for the East China. Sea, Ministry of Agriculture, Xiamen 361021, China

* Corresponding authors:

Chaotian Xie.

Email: ctxie@jmu.edu.cn

Address: Fisheries College, Jimei University, Xiamen 361021, China

Wenlei Wang

Email: wlwang@jmu.edu.cn

Address: Fisheries College, Jimei University, Xiamen 361021, China

#These authors contributed equally to this work.

Supplementary Figures

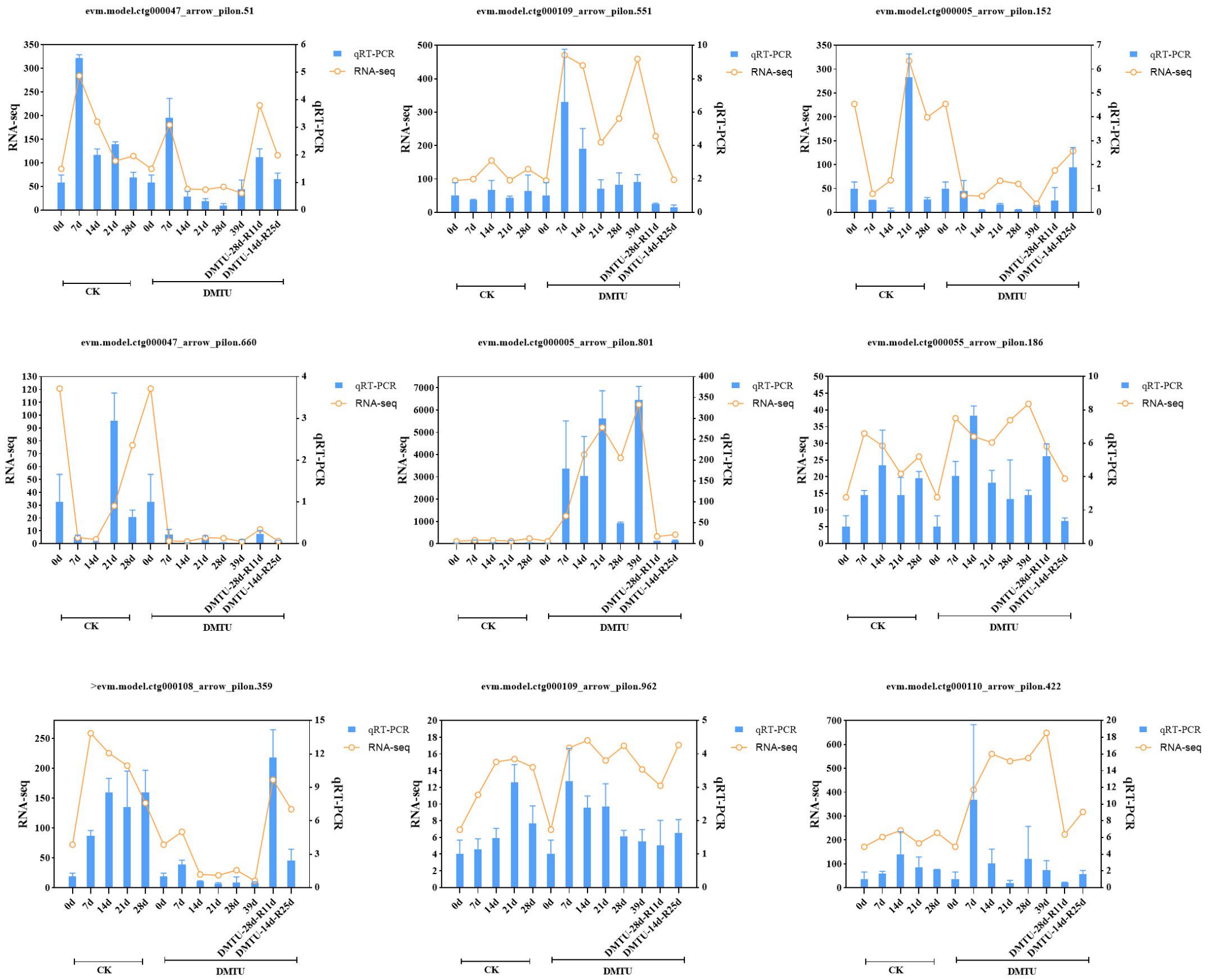

Supplementary fig. 1 qPCR validation of nine genes in the transcriptome of *Pyropia haitanensis* under different maturation-inducing conditions

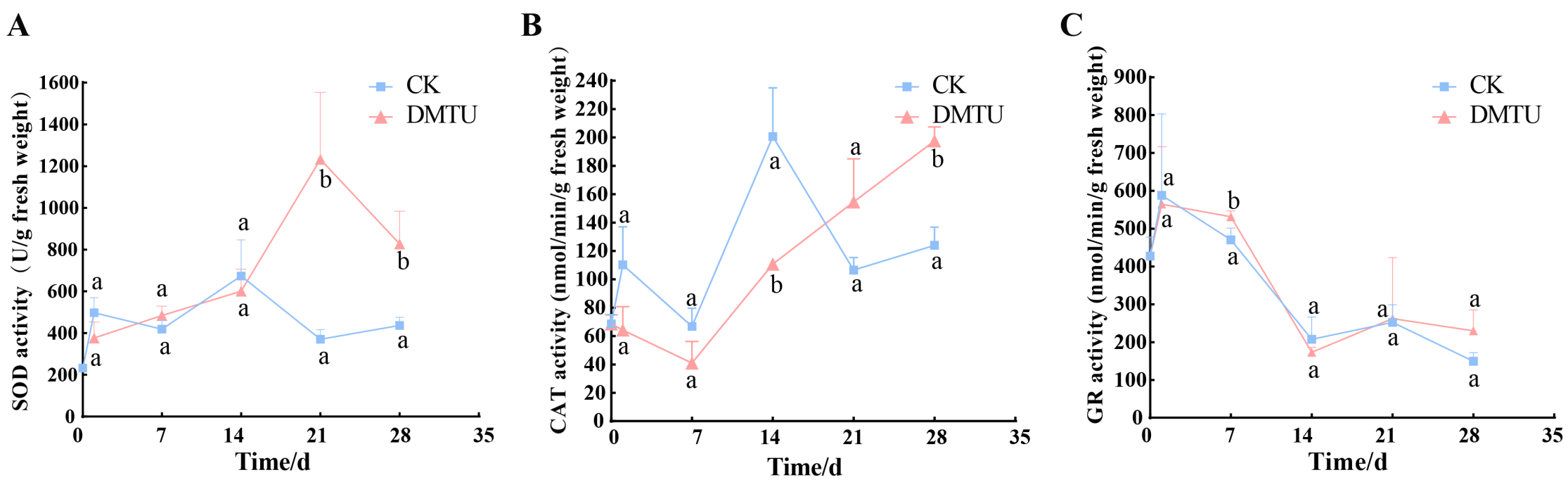

Supplementary fig. 2 SOD enzyme activity (A) ,CAT enzyme activity (B) and GR enzyme activity (C)

Note: Different lowercase letters (a, b, c) represent significant differences among treatment groups at the same time point ($P < 0.05$), whereas identical letters indicate no significant difference.

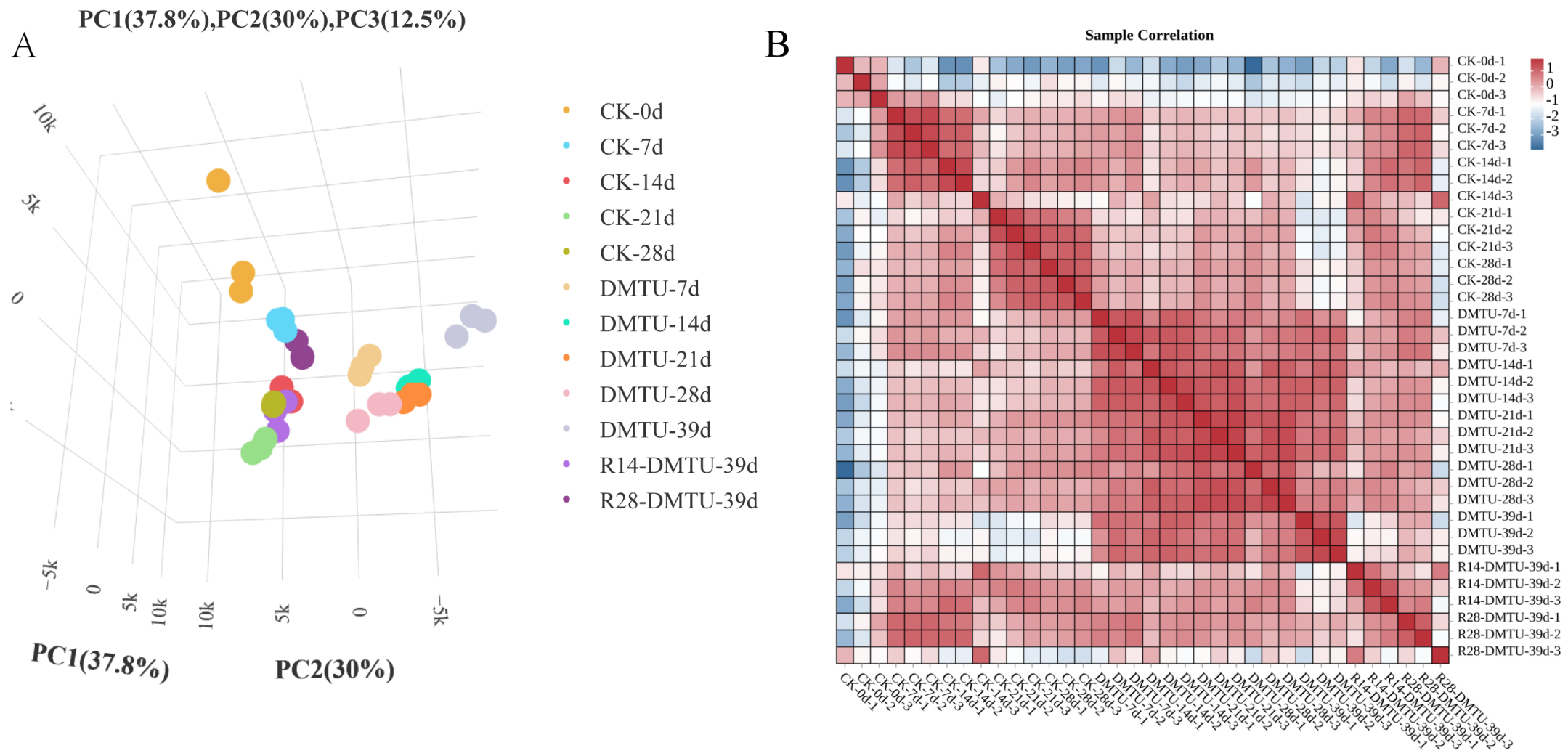

Supplementary fig. 3 Transcriptome principal component analysis (A) and sample correlation analysis (B)

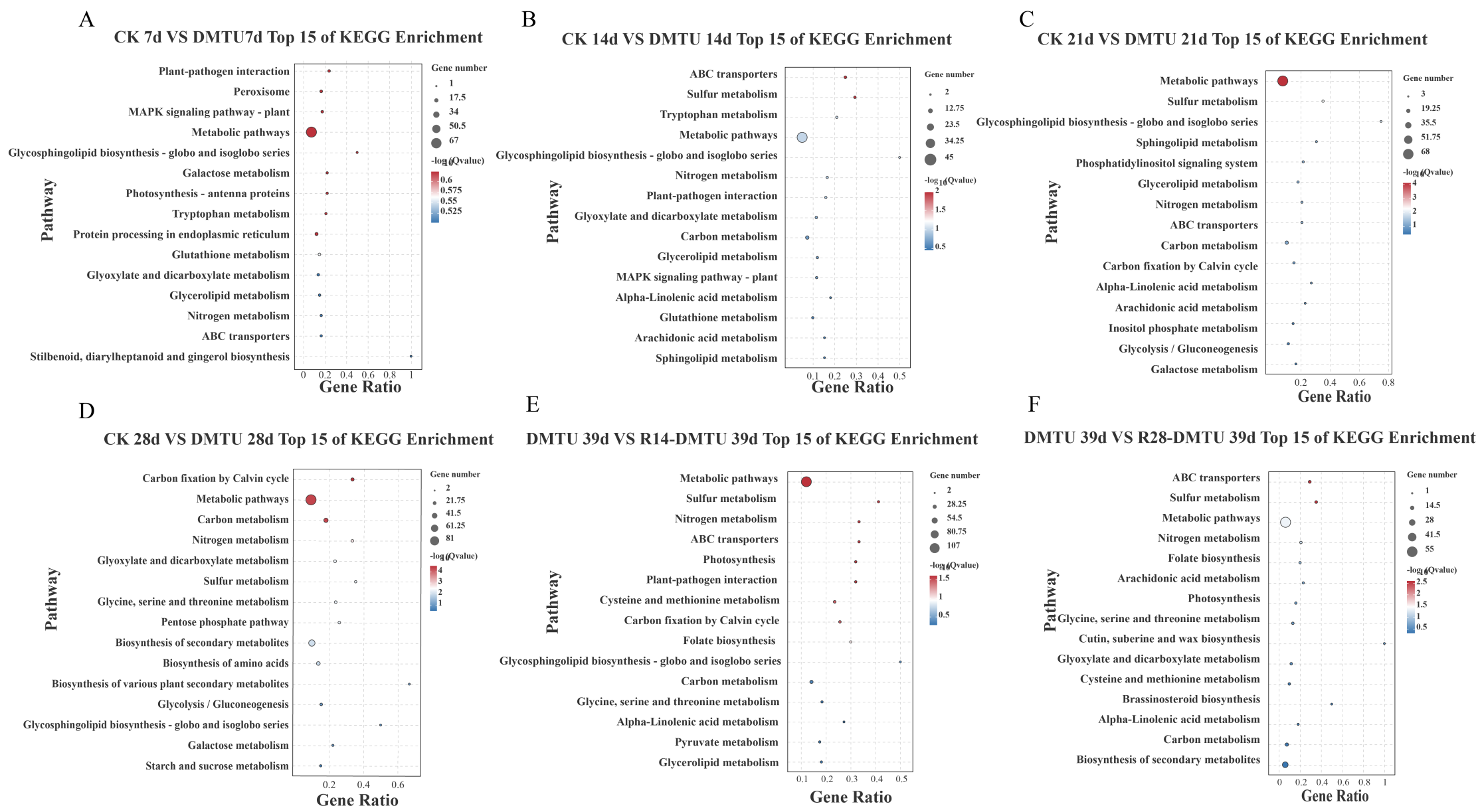

Supplementary fig. 4 KEGG enrichment analysis of differentially expressed genes

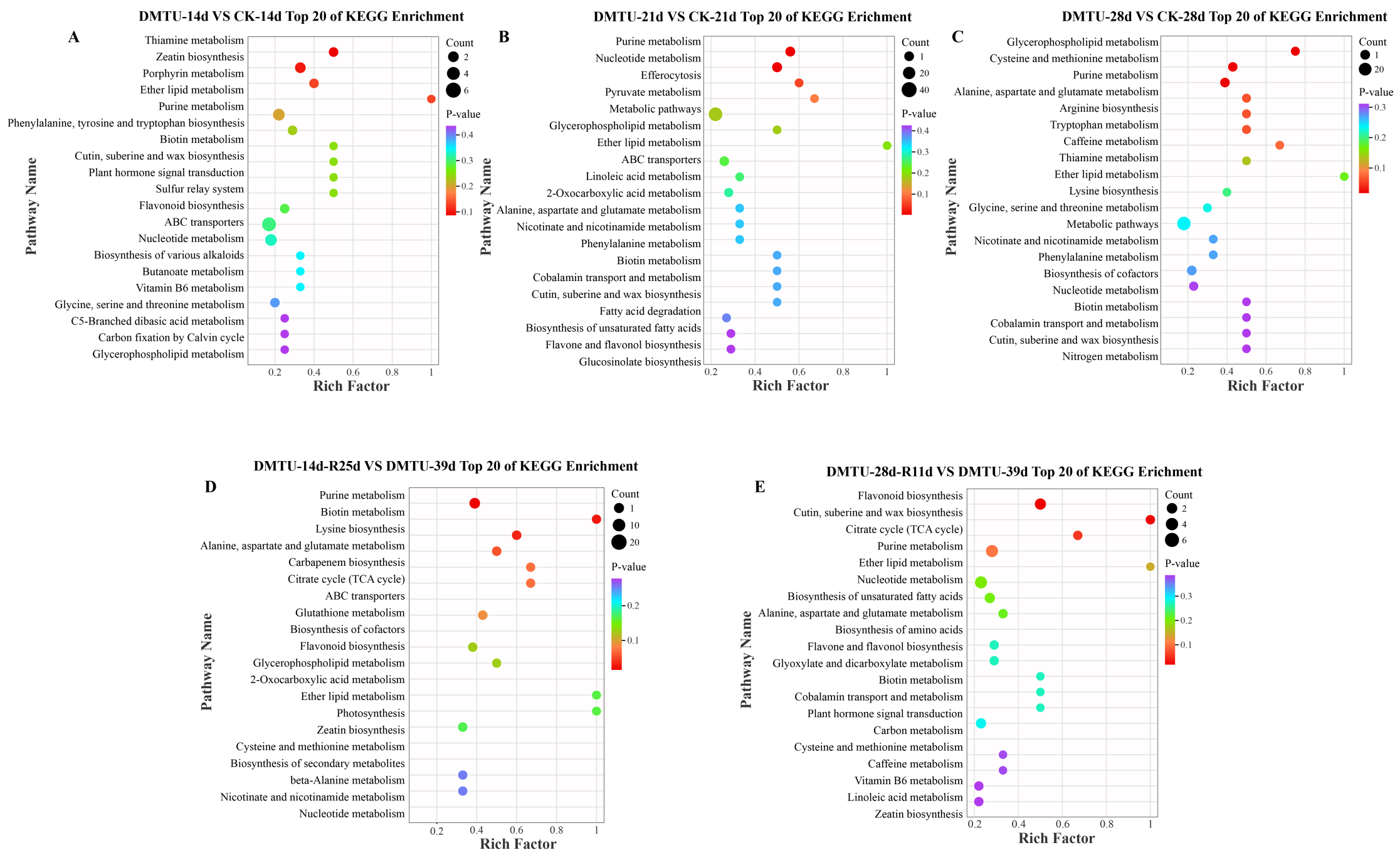

Supplementary fig. 5 Analysis of KEGG enrichment of differential metabolites

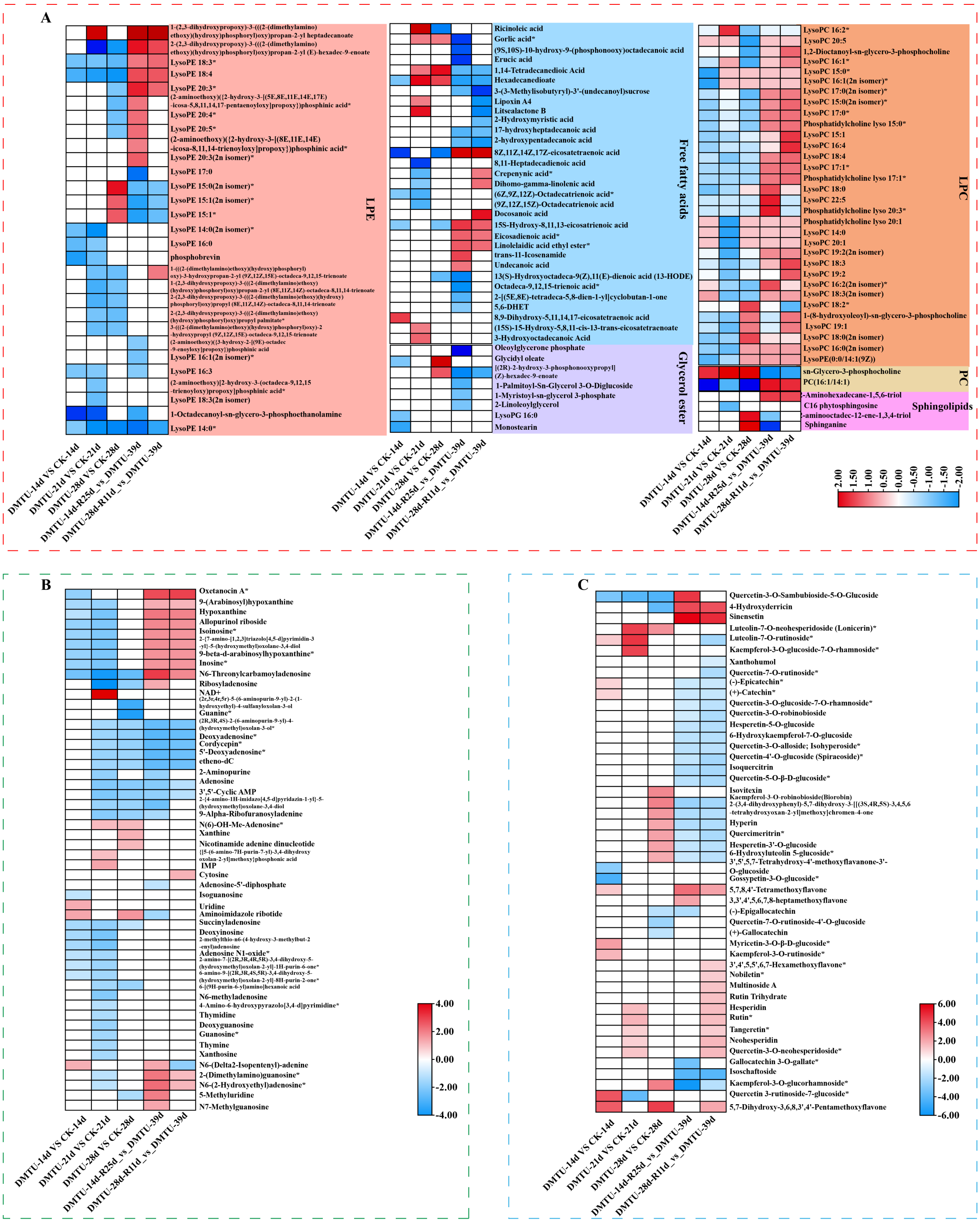

Supplementary fig. 6 Analysis of differential lipid (A) , differential nucleotide (B) and differential isoflavone (C) expression patterns

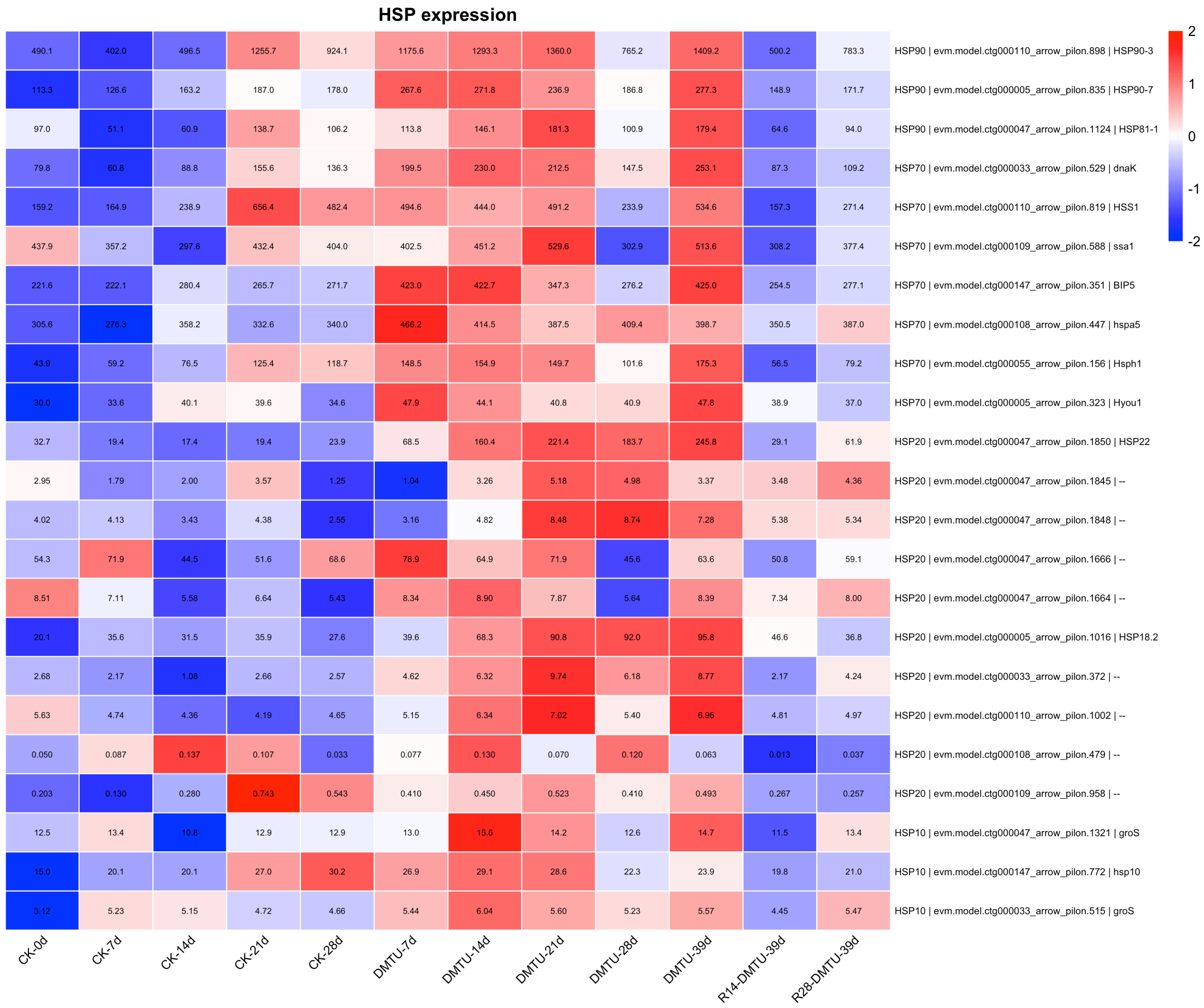

Supplementary Fig. 7. Expression patterns of major HSP gene families under different treatments

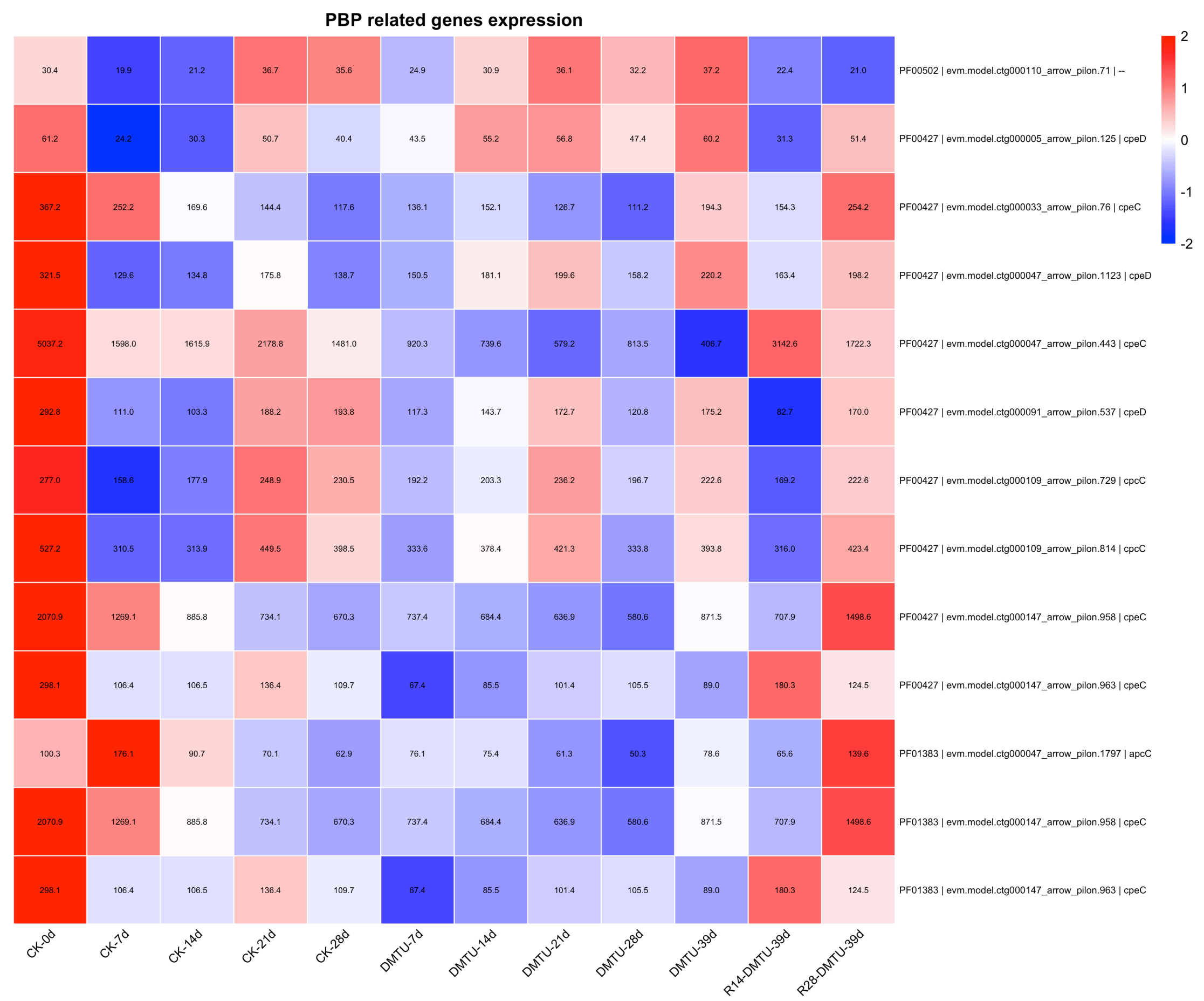

Supplementary Fig. 8. Expression patterns of phycobilisome-related genes under different treatments.

Supplementary table

Supplementary. Tab. 1. The name and sequence of pimers used in the transcriptomes

Gene Id	Gene	Primer Sequence
evm.model.ctg000047_arrow_pilon.51	NRT2.5	Primer F:CGTCTTCCTGGTGGGCACTG<br>Primer R:AGCCTCGGTGACCACAAACG
evm.model.ctg000109_arrow_pilon.551	noxA	Primer F:TCATGATCCACGGCCTGCAC<br>Primer R:ACCTCCATCCGCACCTCCTT
evm.model.ctg000005_arrow_pilon.152	PGK	Primer F:CTGCATTGGTGACGGCGTTG<br>Primer R:CCGCCAGCTTCTTCGCAAAC
evm.model.ctg000047_arrow_pilon.660	ACA3	Primer F:CACCAACTCGCCGTGGAACT<br>Primer R:ACCACGTTGATGGGCCACTG

evm.model.ctg000005_arrow_pilon.801	Hpgds	Primer F:GGGACCTCACCAAGGTGCTC<br>Primer R:ATCGGCGATGGTCAGCTCAG
evm.model.ctg000055_arrow_pilon.186	plcd3a	Primer F:AGCTCGCACAACACGTACC<br>Primer R:TCAATCTCCACACATCGGC
evm.model.ctg000108_arrow_pilon.359	NIA1	Primer F:TGCACTTTGTGCGCAACCAC<br>Primer R:CACCGGGATGGTGACACTCG
evm.model.ctg000109_arrow_pilon.962	DGK6	Primer F:GGTGGGCTCCAAGTTTACCA<br>Primer R:GCCATACGACGGGATGTTGA
evm.model.ctg000110_arrow_pilon.422	Abcb6	Primer F:CACCAACTCGCCGTGGAACT<br>Primer R:ACCACGTTGATGGGCGACTG
UBC		Primer F:TCACAACGAGGATTTACCACC<br>Primer R:GAGGAGCACCTTGGAAACG

Supplementary. Tab. 2: Abbreviations used in the mechanism figure

Abbreviation	English full name
AMT	Ammonium transporter
AMY	alpha-amylase
APR	adenylyl-sulfate reductase (glutathione)
APS	Adenosine 5'-Phosphosulfate
ATPS	ATP sulfurylase
Cys	Cysteine
CysE	Serine O-acetyltransferase
CysK	cysteine synthase
DG	Diacylglycerol
DGK	Diacylglycerol kinase
DMTU	Dimethylthiourea
Gln	Glutamine
Glu	Glutamate
HemA	Glutamyl-tRNA reductase
HSP	Heat shock protein
I4P	myo-Inositol 4-phosphate
IP2	Inositol 1,4-bisphosphate
IP3	1,4,5-Inositol trisphosphate
LHC	Light-harvesting complex
NiR	Nitrite reductase
NR	Nitrate reductase
NRT	Nitrate transporter
OAS	O-Acetylserine
petC	cytochrome b6-f complex iron-sulfur subunit
PI	Phosphatidylinositol
PI(3)P	Phosphatidylinositol Trisphosphate
PI(4)P	Phosphatidylinositol 4-phosphate
PI(4,5)P2	Phosphatidylinositol-4,5-bisphosphate
PIK3C3	phosphatidylinositol 3-kinase
PLC	Phospholipase C

Abbreviation	English full name
Psb27	Photosystem II Psb27 protein
PSI	Photosystem I
PSII	Photosystem II
Psub	photosystem II PsbU protein
SAL	inositol polyphosphate 1-phosphatase
Ser	Serine
SiR	Sulfite reductase
TCA Cycle	Tricarboxylic Acid Cycle
